# Molecular Phylogeny and Morphology Reveal Four New Species of *Conocybe* (Bolbitiaceae, Agaricales) from the Qinghai-Xizang Plateau, China

**DOI:** 10.3390/jof11010045

**Published:** 2025-01-07

**Authors:** Xi-Xi Han, Dorji Phurbu, Bin Cao, Jia-Xin Li, Xin-Yu Zhu, Lin-Hui Liu, Naritsada Thongklang, Kevin D. Hyde, Rui-Lin Zhao

**Affiliations:** 1 Tibet Plateau Key Laboratory of Mycology, Tibet Plateau Institute of Biology, Lhasa 850000, China; 2 School of Science, Mae Fah Luang University, Chiang Rai 57100, Thailand; 3 Center of Excellence in Fungal Research, Mae Fah Luang University, Chiang Rai 57100, Thailand; 4 State Key Laboratory of Mycology, Institute of Microbiology, Chinese Academy of Sciences, Beijing 100101, China; 5 College of Life Sciences, University of Chinese Academy of Sciences, Beijing 100049, China

**Keywords:** Bolbitiaceae, multigene, morphology, phylogeny, Qinghai-Xizang Plateau

## Abstract

The Qinghai-Xizang Plateau, known for its high altitude, geological history of plate collision, crustal uplift, and special ecology factors, provides an ideal environment for studying fungal biodiversity in extreme environmental conditions. Some species within the *Conocybe*, containing secondary metabolites such as psilocybin, phallotoxins, and amatoxins, have potential medicinal value for treating psychiatric disorders and for use in drug development. This study investigates *Conocybe* (Bolbitiaceae, Agaricales) on the Plateau, based on specimens collected over the past decade, using morphological and molecular phylogenetic analyses. Seven species were identified, including four new species: *C. alticola*, *C. alticoprophila*, *C. versicolor*, and *C. yadongensis*. Molecular analyses, utilizing multi-gene sequence data (ITS, nrLSU, and *tef-1α*), support the taxonomic position of these new species within this genus as new species. Detailed descriptions, illustrations, photographs, line drawings, and comparisons with related species are provided for the new taxa. This study enriches the species diversity of *Conocybe* on the Qinghai-Tibet Plateau, further enhancing our understanding of fungal biodiversity in this region.

## 1. Introduction

The Qinghai-Xizang Plateau (abbreviation Q-X Plateau), with an average elevation exceeding 4000 m, and referred to as the “roof” of the world, is the highest and youngest plateau on earth [1]. It is known for its complex geological evolution, including plate collisions, crustal uplift, diverse climatic conditions, and unique ecological features [2,3,4,5,6]. As an “ecological island” isolated from the surrounding lower regions, the plateau has fostered a distinctive high-altitude biogeographic system [7,8]. These exceptional natural conditions have not only promoted the formation of rich and unique biodiversity in the region, but also made it an invaluable resource for global scientific research [9,10,11]. The region’s diverse plant communities and unique environmental conditions provide an ideal natural platform for studying fungal adaptive evolution, making the Q-X Plateau a critical area for exploring high-altitude ecosystems and fungal biodiversity [12,13,14]. Due to challenges posed by remote location, limited accessibility and harsh climatic conditions, research on fungal communities in this region has progressed slowly, leaving numerous valuable fungal resources yet to be explored [15,16].

*Conocybe* Fayod belongs to Bolbitiaceae (Agaricales) [17], is characterized by its small to medium-sized delicate basidiomata, conical pileus, adnexed to adnate and brown-rusty lamellae, powdery or pilose stipes, lecythiform cheilocystidia, and basidiospores with germ pore [18,19]. Most *Conocybe* species are saprophytic and are widely distributed in different vegetation types, particularly in fertile soils and herbivore dung, as well as on plant debris, decaying wood, and sawdust [18,20,21,22]. The genus *Conocybe* is noted for its chemical diversity and biological activities, despite some species containing neurotoxic or cytotoxic substances [23,24,25,26]. It has been believed that secondary metabolites like psilocybin, with potential therapeutic effects, and toxic cyclic peptides such as amatoxins and phallotoxins may cause severe health issues [27,28,29,30].

*Conocybe* is divided into more than 10 sections based on morphological characteristics, including *sect. Conocybe* Fayod, *sect. Mixtae* Singer, *sect. Candidae* Watling, and *sect. Pilosellae* Singer [18,31,32,33,34]. Currently, approximately 270 species of *Conocybe* have been identified worldwide, with 44 recorded in China [18,19,35,36,37]. Recent studies have focused on *Conocybe* specimens from various regions in China. Song and Bau (2023) described seven new species and one new record from *sect. Pilosellae* collected in northeastern, northern, and central–southern China [18]. In 2024, Wang et al. described *C*. *himalayana* Ke Wang, T.Z. Wei & P. Hong from *sect. Pilosellae* based on five specimens collected from the Q-X Plateau [16], and Lu et al. reported two new species from *sect. Conocybe* based on four specimens from the Yunnan-Guizhou Plateau [36]. However, research on *Conocybe* in the Q-X Plateau remains limited. This study investigates *Conocybe* specimens collected over the past decade from the plateau, combining macroscopic, microscopic, and molecular methods to explore species diversity and distribution in the region.

## 2. Materials and Methods

### 2.1. Samplings and Morphological Study

The specimens for this study were collected from Lhasa Municipality, Qamdo Municipality, Shigatse Municipality, Lingzhi Prefecture, and the Ali Region in the Xizang Autonomous Region in China between 2015 and 2024. Photographs were captured using a Canon EOS 80D camera (Tokyo, Japan), and specimens were carefully packed individually in aluminum foil to prevent mixing or damage. Specimens were dried completely using a food dryer at 50 °C, sealed in plastic bags, and deposited in the Mycological Herbarium of the Institute of Microbiology, Chinese Academy of Sciences (HMAS). Microscopic features such as basidia, basidiospores, pileipellis, and cheilocystidia were observed under an Olympus CX31 light microscope (Tokyo, Japan), with at least 30 measurements obtained for each feature. Microscopic examination followed the methodology of Largent [38]. Tissues were treated with 5% KOH and sterile water for observation. Measurements are presented as (a)b–c(d), where b–c represents at least 90% of the measured values, while a and d indicate the lowest value and the highest value, respectively. The Q value is the length/width ratio of basidiospores, with Qm representing the mean Q value ± standard deviation [39]. The color designations were determined using the *Methuen Handbook of Colour* [40].

### 2.2. DNA Extraction, PCR and Sequencing

DNA was extracted from dried specimens using the Broad-spectrum Plant Rapid Genomic DNA Kit (Biomed, Beijing, China), following the manufacturer’s instructions. The primer pairs ITS1F/ITS4 [41], LR7/LR0R [42], and EF983F/EF2218R [43] were used to amplify the nuclear internal transcribed spacer (ITS), the large subunit nuclear ribosomal DNA (nrLSU), and the translation elongation factor subunit 1 alpha (*tef-1α*) regions, respectively. The polymerase chain reaction (PCR) procedure was performed under the following conditions: an initial denaturation at 94 °C for 5 min, followed by 35 cycles of denaturation at 94 °C for 60 s, annealing at 53 °C (ITS), 50 °C (nrLSU), or 55 °C (*tef-1α*) for 60 s, and extension at 72 °C for 90 s, with a final extension at 72 °C for 10 min [44,45,46]. The PCR products were detected by electrophoresis and sent to BGI Genomics Co., Ltd. (Beijing, China) for purification and sequencing.

### 2.3. Phylogenetic Analyses

Based on BLAST searches against GenBank and previous studies, we analyzed the nrITS, nrLSU and *tef-1α* sequences of 124 taxa, as detailed in Table 1. The sequences were aligned using Muscle version 3.6 separately [47], then manually adjusted in BioEdit version 7.0.4 to remove the ambiguous areas [48]. The aligned sequences were assembled in PhyloSuite version 1.2.3 [49]. Maximum likelihood (ML) analysis was performed on the concatenated sequences using raxmlGUI 1.3 with a GTRGAMMA model and 1000 rapid bootstrap replicates [50]. The best partitioning scheme and evolutionary models for three predefined partitions were selected using PartitionFinder2 v2.1.1 [51], based on the greedy algorithm and AICc criterion: GTR+I+G for ITS, GTR+I+G for nrLSU, and SYM+I+G for *tef-1α*. Bayesian Inference (BI) analysis was conducted using MrBayes v3.2.7a [52], with six Markov chains run for two million generations and trees sampled every 100th generation. The burn-ins were determined using Tracer version 1.6, with an ESS value higher than 200. The remaining trees were used to calculate Bayesian posterior probabilities (PP). The trees were displayed in Interactive Tree of Life (iTOL) version 6 [53].

## 3. Results

### 3.1. Phylogeny

**Figure 1:**
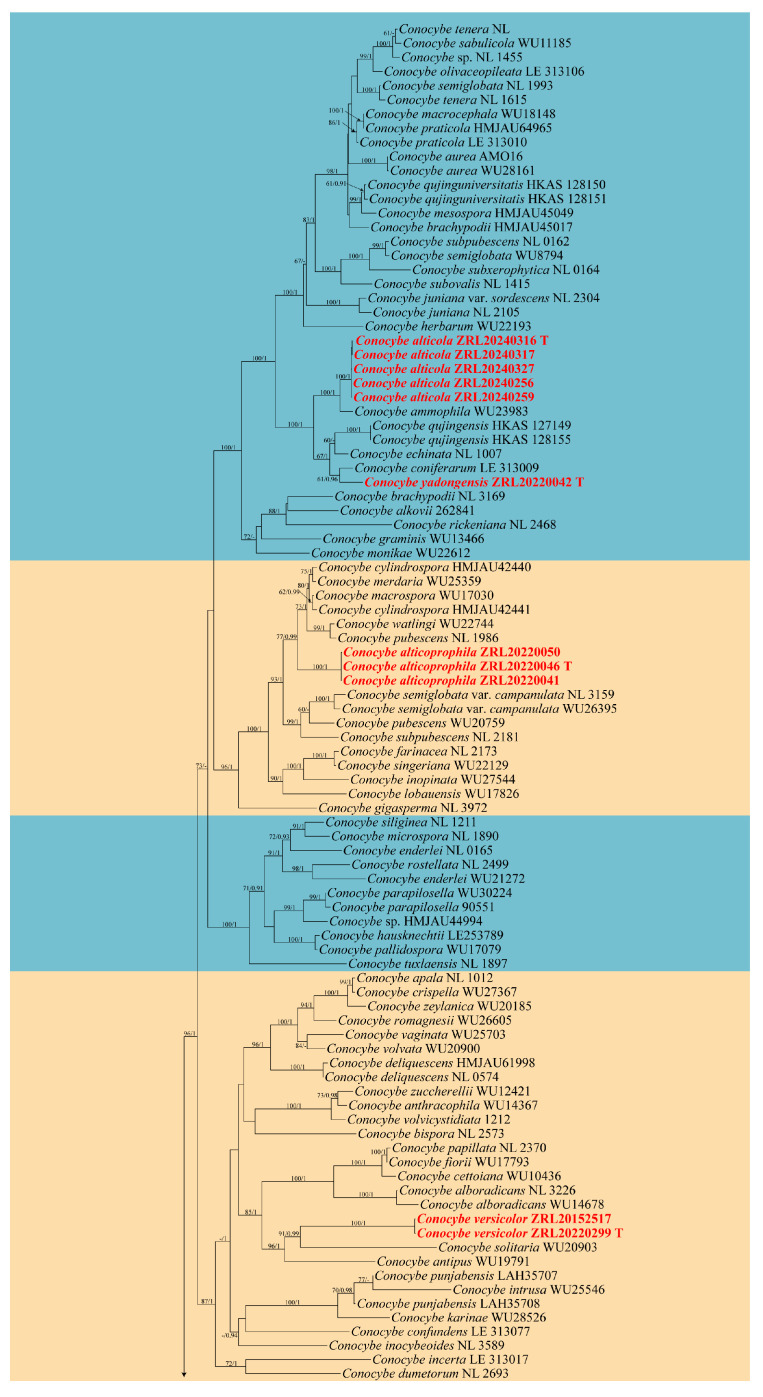

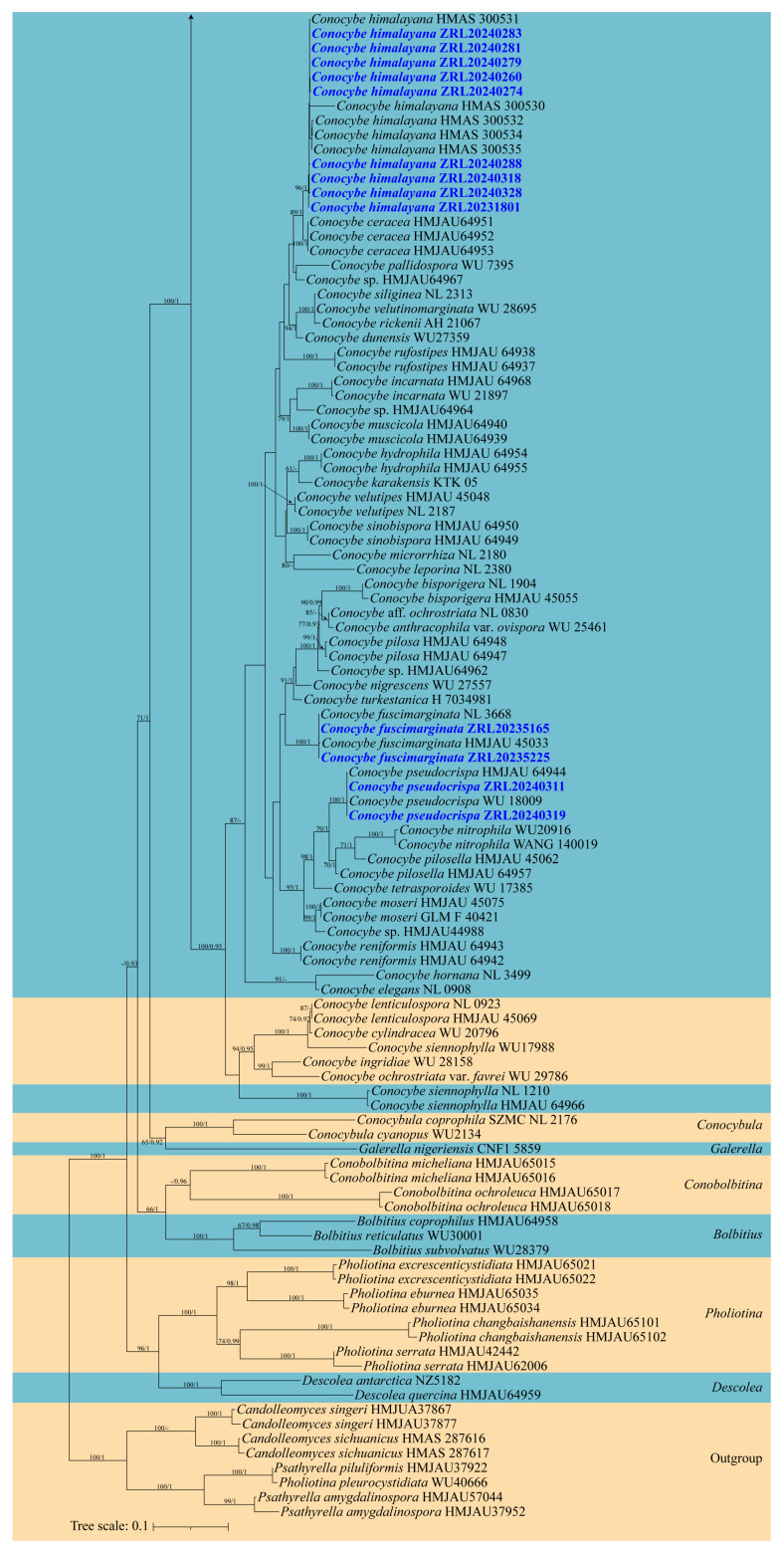
Molecular phylogenetic analyses of *Conocybe* species by the maximum likelihood (ML) method based on combined nrITS, nrLSU and *tef-1α* sequences. Maximum likelihood bootstrap support values (ML) ≥ 60% and Bayesian posterior probabilities (PP) ≥ 0.90 are shown at the nodes as ML/PP. Sequences newly generated in this study are highlighted in colored font.

In the phylogenetic analysis, 24 specimens from seven *Conocybe* species were included, with the following four species from the *Psathyrellaceae* Vilgalys, Moncalvo & Redhead selected as outgroups: *Psathyrella piluliformis* (Bull.) P.D. Orton, *P. amygdalinospora* T. Bau & J.Q. Yan, *Candolleomyces sichuanicus* R.L. Zhao, B. Cao & X.X. Han, and *C. singeri* (A.H. Sm.) D. Wächt. & A. Melzer. Furthermore, it is noteworthy that Song (2024), through morphological observations and sequence comparisons, proposes that *Pholiotina pleurocystidiata* Hauskn. & Krisai should be considered a synonym of *Psathyrella piluliformis* [62,64]. In total, 72 new sequences were generated in this study, which were from 24 specimens from Qinghai-Xizang Plateau, China, all with the nrITS, nrLSU, and *tef-1α* sequences. The combined dataset with 3310 characters including gaps (864 for nrITS, 1299 for nrLSU, and 1147 for *tef-1α*) was included in the phylogenetic analyses. The phylogenetic trees of ML and MrBayes were almost identical. The ML tree is shown in Figure 1 with bootstrap values and Bayesian posterior probabilities indicated on the branches.

### 3.2. Taxonomy

**Figure 2:**
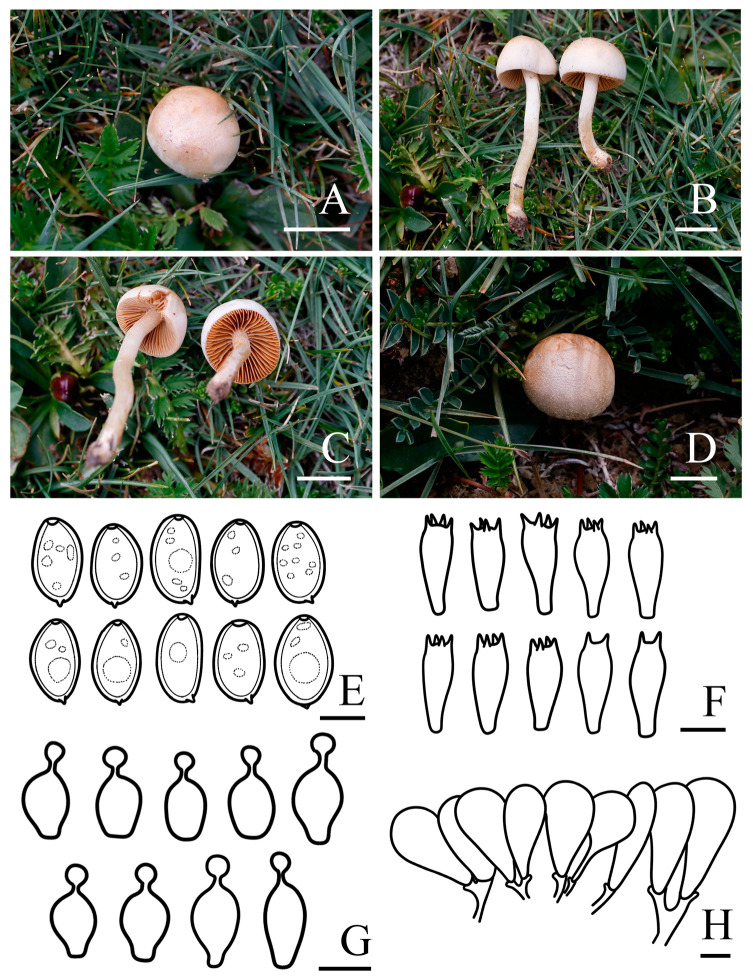
Basidiomata and microscopic features of *Conocybe alticola.* (**A**–**D**) Basidiomata: (**A**–**C**) ZRL20240316 (holotype); (**D**) ZRL20240317, (**E**) Basidiospores, (**F**) Basidia, (**G**) Cheilocystidia, (**H**) Pileipellis. Scale bars: 10 mm (**A**–**D**); 5 μm (**E**); 10 μm (**F**–**H**).

***Conocybe alticola*** R.L. Zhao & X.X. Han, sp. nov., Figure 2

*Fungal Names*: FN 572250

*Holotype*: China. Xizang Autonomous Region, Ali Region, Zhada County, Rural Road 748, Qinipu, N 31°45′43″ E 79°32′49″, 4058 m asl, 5 August 2024, *Xi-Xi Han*, *Lin-Hui Liu* ZRL20240316 (**holotype** HMAS 287991). GenBank: PQ699270 (nrITS), PQ699294 (nrLSU), PQ836632 (*tef-1α*).

*Etymology: alticola* (Latin) refers to the habitat of the species, which is adapted to high-altitude environments.

*Diagnosis: Conocybe alticola* is distinguished by its hemispherical, obtusely conical to convex pileus with a non-striate surface and a pale yellow hue, the stipe slightly enlarged at base, sometimes forming a bulb. The narrowly ellipsoid to oblong basidiospores, with a germ pore. Grows on alpine meadows in summer.

*Macroscopic description:* **Pileus** 1.2–2.0 cm diam., hemispherical, obtusely conical to convex, not hygrophanous, faintly pubescent, non-striate, edge decurved, sometimes with slightly undulated edge, carnosus; yellowish white (4A2) to greyish yellow (4B3), blond (4C4) to olive brown (4D6) at the center, edge paler, pruinose surface. **Context** thin, fleshy, yellowish white (4A2) to blond (4C4), indistinct odor. **Lamellae** are distant to nearly distant, narrowly adnate to adnexed, unequal in length, orange–white (5A2) to greyish yellow (4B3) with pale edge when young, light brown (6D7) when mature. **Stipes** 1.3–3.8 × 0.2–0.3 cm, cylindrical, slightly enlarged at base, sometimes forming a bulb, slightly longitudinally striated, with blond (4C4) pruinose, yellowish white (4A2) to greyish yellow (4C5). **Odor** not distinctive. **Taste** indistinct.

*Microscopic description:* **Basidiospores** (9.4)9.5–10.5(11.1) × (5.5)6.0–6.7(7.1) μm, Q = 1.48–1.70, Qm = 1.59 (±0.11), narrowly ellipsoid to oblong, with germ pore, slightly thick-walled, contains oil droplets, pale yellow (4A3) to greyish yellow (4C6) in water, greyish orange (6B3) to light brown (6D7) in 5% KOH. **Basidia** (23.5)24.5–27.1(28.5) × (8.1)9.0–10.0(10.4) μm, clavate, sometimes with vacuolar contents, two or four spored. **Cheilocystidia** (14.5)16.7–21.2(25.2) × (6.9)8.0–9.8(10.7) μm, lecythiform, with 3.9–4.6 μm wide capitula. **Pileipellis** hymeniform, consist of broadly clavate, obovoid, or spheropedunculate elements, (25.9)29.1–42.7(54.2) × (10.2)12.3–18.8(20.4) μm. **Pleurocystidia** clavate or lecythiform, and rare in number. **Pileocystidia** not observed. **Clamp connections** present.

*Habit and habitat:* Summer solitary or scattered in the alpine meadows. So far found only in the Xizang Autonomous Region, China.

*Other specimens examined*: China. Xizang Autonomous Region, Ali Region, Gaer County, Zuozuo Township, N 32°23′55″ E 80°14′59″, 4409 m asl, 31 July 2024, *Xi-Xi Han*, *Lin-Hui Liu* ZRL20240256 (HMAS 287982); Xizang Autonomous Region, Ali Region, Gaer County, Zuozuo Township, N 32°23′55″ E 80°14′59″, 4409 m asl, 31 July 2024, *Xi-Xi Han*, *Lin-Hui Liu* ZRL20240259 (HMAS 287983); Xizang Autonomous Region, Ali Region, Zhada County, Rural Road 748, Qinipu, N 31°45′43″ E 79°32′49″, 4058 m asl, 5 August 2024, *Xi-Xi Han*, *Lin-Hui Liu* ZRL20240317 (HMAS 287992); Xizang Autonomous Region, Ali Region, Zhada County, Paerlong, N 31°15′39″ E 79°54′16″, 4179 m asl, 6 August 2024, *Xi-Xi Han*, *Lin-Hui Liu* ZRL20240327 (HMAS 287995).

*Notes:* Phylogenetically and morphologically, the new species *C. alticola* is closely related to *C. ammophila* (Figure 1) [67], but can be distinguished by its hemispherical, obtusely conical to convex, non-striate, carnosus pileus; pale yellow hue stipe; narrower, greyish orange to light brown in 5% KOH basidiospores and two or four-spored basidia, while *C. ammophila*, originally described from Greenland, has a pale stipe, broader spores, and four-spored basidia [67,68]. *Conocybe alticola* is widely distributed in the alpine and subalpine regions of the southwestern Q-X Plateau.

**Figure 3:**
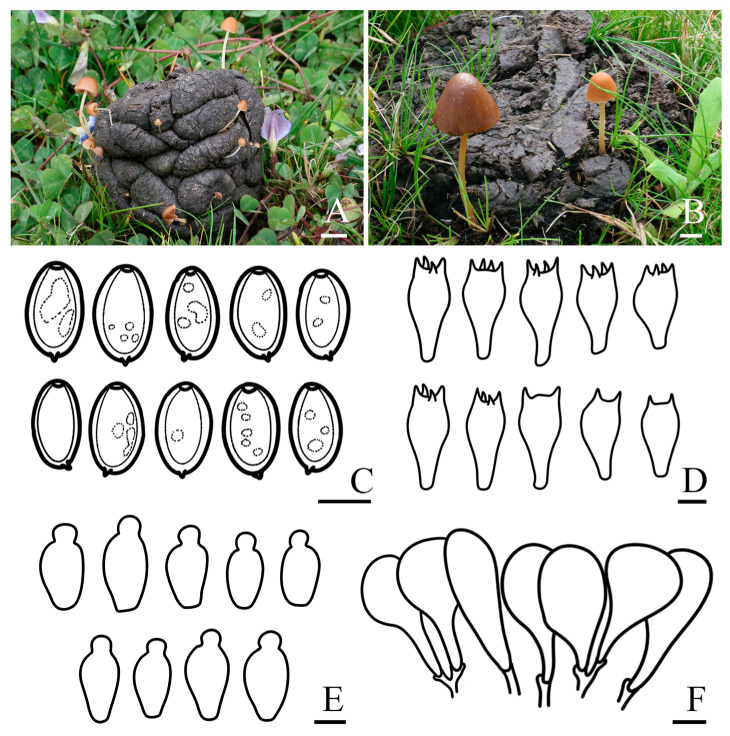
Basidiomata and microscopic features of *Conocybe alticoprophila.* (**A**,**B**) Basidiomata: (**A**) ZRL20220041; (**B**) ZRL20220046 (holotype), (**C**) Basidiospores, (**D**) Basidia, (**E**) Cheilocystidia, (**F**) Pileipellis. Scale bars: 10 mm (**A**,**B**); 10 μm (**C**–**F**).

***Conocybe alticoprophila*** R.L. Zhao & X.X. Han, sp. nov., Figure 3

*Fungal Names*: FN 572251

*Holotype*: China. Xizang Autonomous Region, Shigatse Municipality, Yadong County, Lower Yadong Township, N 27°13′12″ E 88°34′48″, 2872 m asl, 26 July 2022, *Rui-Lin Zhao*, *Bin Cao* ZRL20220046 (holotype HMAS 287975). GenBank: PQ699274 (nrITS), PQ699298 (nrLSU), PQ836636 (*tef-1α*).

*Etymology:* “alti-” derived from the Latin word “*altus*”, meaning “high-altitude”; “-coprophila” derived from the Greek words “*copros*” and “*philos*”, meaning “dung” and “fondness”, respectively.

*Diagnosis: Conocybe alticoprophila* is characterized by its small to medium-sized basidiomata, yellowish-brown, hygrophanous pileus, which is translucently striate to 4/5 from the edge to the center. The basidiospores are slightly amygdaliform, and basidia are two or four spored. Grows on cow dung in grasslands.

*Macroscopic description:* **Pileus** 0.2–3.0 cm diam., obtusely conical, hemispherical to conical–convex, hygrophanous, translucently striate to 4/5 from the edge to the center when moist, surface smooth, edge decurved, sometimes with slightly undulated edge; greyish orange (5B4) to yellowish brown (5E5) at the center, turning orange–white (5A2) to yellowish brown (5D8) towards edge. **Context** thin, and indistinct odor. **Lamellae** are moderately crowded, narrowly adnate to adnexed, orange–white (5A2) to yellowish brown (5D5). **Stipes** 0.8–5.2 × 0.1–0.3 cm, cylindrical, fragile, slightly enlarged at the base forming a weak bulb, slightly longitudinally striate, with orange–white (5A2) pruinose, orange–white (5A1) to brownish orange (5C5). **Odor** not distinctive. **Taste** indistinct.

*Microscopic description:* **Basidiospores** (15.9)16.4–17.7(18.0) × (9.7)10.0–11.0(12.0) μm, Q = 1.55–1.71, Qm = 1.63 (±0.08), narrowly ellipsoid to oblong, slightly amygdaliform, with germ pore, thick-walled, contains oil droplets, pale orange (5A3) to brownish yellow (5C7) in water, light brown (6D6) to dark brown (6F7) in 5% KOH. **Basidia** (26.3)27.1–31.4(33.5) × (14.2)14.7–15.8(16.6) μm, clavate to broadly clavate, sometimes with vacuolar contents, two or four spored. **Cheilocystidia** (19.7)21.5–27.0(30.7) × (11.2)12.2–15.5(17.5) μm, lecythiform, with 4.7–7.0 μm wide capitula. **Pileipellis** hymeniform, consist of clavate or spheropedunculate elements, (26.2)27.6–44.0(47.3) × (10.5)12.9–22.0(24.0) μm. **Pleurocystidia** lecythiform, and rare in number. **Pileocystidia** not observed. **Clamp connections** present.

*Habit and habitat:* Summer solitary or scattered on cow dung in grasslands. So far found only in the Xizang Autonomous Region, China.

*Other specimens examined*: China. Xizang Autonomous Region, Shigatse Municipality, Yadong County, Lower Yadong Township, N 27°13′12″ E 88°34′48″, 2872 m asl, 26 July 2022, *Rui-Lin Zhao*, *Bin Cao* ZRL20220041 (HMAS 287973); Xizang Autonomous Region, Shigatse Municipality, Yadong County, Lower Yadong Township, N 27°13′12″ E 88°34′48″, 2872 m asl, 26 July 2022, *Rui-Lin Zhao*, *Bin Cao* ZRL20220050 (HMAS 287976).

*Notes: C. alticoprophila* is a coprophilous species characterized by a small basidioma, conical and yellowish-brown pileus, and a cylindrical stipe with finely pruinose surface, it is similar to *C. pubescens* (Gillet) Kühner [69]. However, *C. pubescens* has a smaller basidiospores and pileus lack translucently striate when moist. The pileus of *C. alticoprophila* is obtusely conical, hemispherical to conical–convex with a cylindrical stipe that is slightly enlarged at the base forming a weak bulb. Morphologically, it resembles *C. watlingii* Hauskn, and is closely related phylogenetically. In contrast, the basidiospores and basidia of *C. watlingii* are narrower, its pileus lacks translucently striate edges when moist, and the cheilocystidia are slightly narrower with smaller capitula [70]. *C. alticoprophila* is phylogenetically and morphologically closely related to *C. cylindrospora* T. Bau & J. Liu, but the latter has smaller basidiospores that are cylindrical to ellipsoid-oblong, four-spored basidia which are also smaller, and smaller cheilocystidia [19]. The *Conocybe* sp. from Uruguay (PP949255 and PP949256) in the NCBI GenBank shows a high sequence similarity with the ITS of *C. alticoprophila*, due to partial sequence gaps and the lack of morphological information for the specimens, we cannot confirm whether they represent the same species.

**Figure 4:**
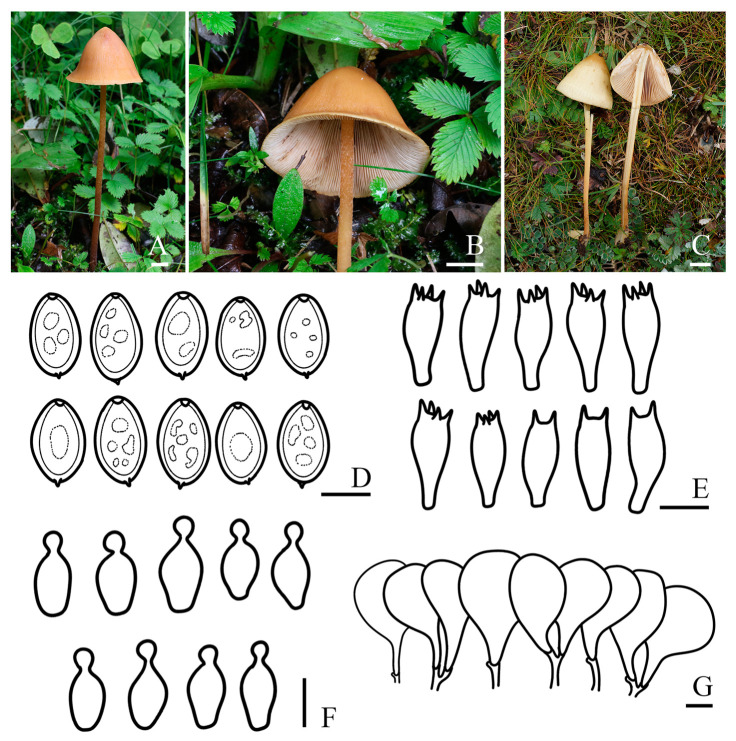
Basidiomata and microscopic features of *Conocybe versicolor.* (**A**–**C**) Basidiomata: (**A**,**B**) ZRL20220299 (holotype); (**C**) ZRL20152517, (**D**) Basidiospores, (**E**) Basidia, (**F**) Cheilocystidia, (**G**) Pileipellis. Scale bars: 10 mm (**A**–**C**); 5 μm (**D**); 10 μm (**E**–**G**).

***Conocybe versicolor*** R.L. Zhao & X.X. Han, sp. nov., Figure 4

*Fungal Names*: FN 572252

*Holotype*: China. Xizang Autonomous Region, Shigatse Municipality, Yadong County, Lower Yadong Township, N 27°25′20″ E 88°55′6″, 3254 m asl, 27 July 2022, *Rui-Lin Zhao*, *Bin Cao* ZRL20220299 (**holotype** HMAS 287978). GenBank: PQ699290 (nrITS), PQ699314 (nrLSU), PQ836639 (*tef-1α*).

*Etymology*: versi- = variable, -color = color, referring to the basidiomata of the species having variable colors.

*Diagnosis:* Pileus surface of *Conocybe versicolor* is smooth with a slightly undulated edge. The stipe is cylindrical, hallow, slightly enlarged at the base, covered with scattered to dense pruinose, and no radicating structures were observed. Basidiospores are ellipsoid to oblong. Grow on the ground in mixed forests.

*Macroscopic description:* **Pileus** 2.9–4.6 cm diam., obtusely conical, conical–convex to campanulate with or without distinct broad umbo, hardly expanding, sometimes wrinkled, edge slightly undulated, slightly hygrophanous, not striate, surface smooth; brownish orange (5C5) to yellowish brown (5D8) at the center, edge orange–white (5A2) to greyish orange (5B4). **Context** thin, yellowish white (3A2) to greyish yellow (3C4), and odor indistinct. **Lamellae** are slightly crowded, narrowly adnate to adnexed, unequal in length, with smooth edges. **Stipes** 9.7–15.1 × 0.2–0.4 cm, cylindrical, hallow, slightly enlarged at the base, with scattered to dense orange–white (5A2) pruinose, and no radicating structures were observed. **Odor** not distinctive. **Taste** indistinct.

*Microscopic description:* **Basidiospores** (8.2)8.4–9.1(9.7) × (4.9)5.4–6.1(6.6) μm, Q = 1.43–1.63, Qm = 1.53 (±0.10), ellipsoid to oblong, slightly angular, with germ pore, thick-walled, contains oil droplets, yellowish white (4A2) to greyish yellow (4B4) in water, brownish orange (6C6) to brown (6E8) in 5% KOH. **Basidia** (18.2)19.7–22.3(23.6) × (7.4)7.8–8.6(9.3) μm, clavate, with vacuolar contents, two or four-spored. **Cheilocystidia** (15.1)16.9–19.6(20.9) × (6.9)7.5–9.0(10.0) μm, lecythiform, with 3.4–4.5 μm wide capitula. **Pileipellis** hymeniform, consist of broadly clavate, obovoid, or spheropedunculate elements, (26.9)33.3–44.9(52.4) × (14.0)17.1–23.2(26.5) μm. **Pleurocystidia** clavate or lecythiform, and rare in number. **Pileocystidia** not observed. **Clamp connections** present.

*Habit and habitat*: Summer solitary on the ground in mixed forests. So far found only in the Xizang Autonomous Region, China.

*Other specimens examined*: China. Xizang Autonomous Region, Lingzhi Prefecture, Lulang Town, N 29°46′18″ E 94°44′12″, 3326 m asl, 22 September 2015, *Sheng-Yu Su* ZRL20152517 (HMAS 287972).

*Notes: Conocybe versicolor* has a conical–convex to campanulate pileus, pale orange to greyish orange, resembling *C. solitaria* K.A. Thomas, Hauskn. & Manim. It shares similar basidiospore size and clavate basidia with *C. solitaria*, but the latter can be distinguished by its decurved pileus margin with faint radial striations, and larger basidia [71]. Additionally, *C. versicolor* has a similar stipe, basidiospores size and two or four-spored basidia to *C. humicola* (Thiers) Hauskn., Krisai & Voglmary, but *C. humicola* differs in having smaller basidiomata, shorter basidia, shorter cheilocystidia and a pileipellis consistsing of roundish-stipitate elements [67]. Phylogenetically, *C. versicolor* is closely related to *C. antipus* (Lasch) Kühner, which was originally described from North America. Both species share similar basidiospore sizes and occasionally wrinkled pileus, but *C. antipus* has slightly larger four-spored basidia, and long radicating stipes up to 40 mm [68,70].

**Figure 5:**
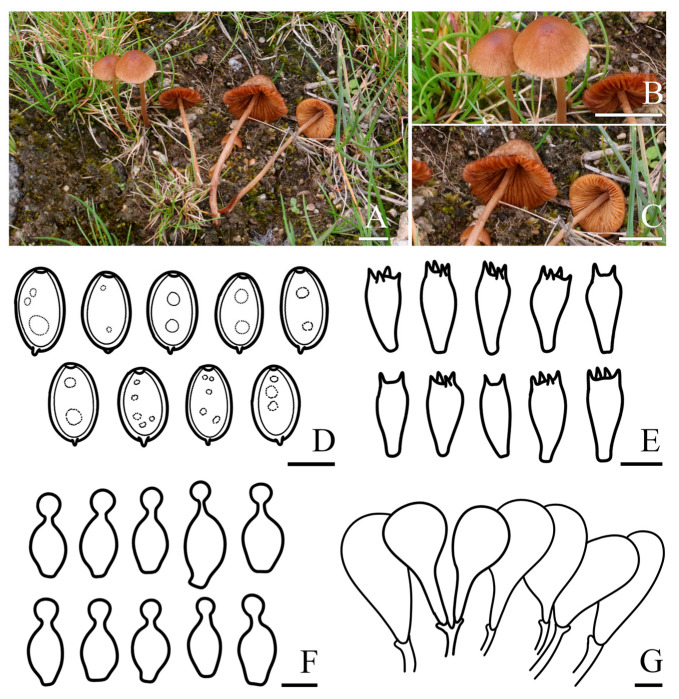
Basidiomata and microscopic features of *Conocybe yadongensis.* (**A**–**C**) Basidiomata ZRL20220042 (holotype), (**D**) Basidiospores, (**E**) Basidia, (**F**) Cheilocystidia, (**G**) Pileipellis. Scale bars: 10 mm (**A**–**C**); 5 μm (**D**); 10 μm (**E**–**G**).

***Conocybe yadongensis*** R.L. Zhao & X.X. Han, sp. nov., Figure 5

*Fungal Names*: FN 572253

*Holotype:* China. Xizang Autonomous Region, Shigatse Municipality, Yadong County, Lower Yadong Township, N 27°13′12″ E 88°34′48″, 2872 m asl, 26 July 2022, *Rui-Lin Zhao*, *Bin Cao ZRL20220042* (**holotype** HMAS 287974). GenBank: PQ699291 (nrITS), PQ699315 (nrLSU), PQ836640 (*tef-1α*).

*Etymology*: *yadongensis* (Latin) refers to Yadong County, Xizang, the locality of the type specimen.

*Diagnosis: Conocybe yadongensis* is characterized by small- to medium-sized basidiomata with a yellowish-brown pileus that is translucently striate and has a surface that is barely pruinose. The stipe is faintly longitudinally striated, and covered with strong pruinosity. The basidiospores are narrowly ellipsoid to oblong. Grows in the grassland.

*Macroscopic description:* **Pileus** 0.7–2.2 cm diam., hemispherical when young, obtusely conical, convex to plano-convex when mature, hygrophanous, translucently striate to 4/5 from the edge to the center when moist, faintly pubescent, surface hardly pruinose, edge slightly undulated; brownish orange (5C5) to yellowish brown (5E8) at the center, edge orange–white (5A2) to brownish orange (5C4). **Context** thin, slightly fragile, orange–white (5A2) to brownish orange (5C5), odor indistinct. **Lamellae** are slightly crowded, narrowly adnate to adnexed, unequal in length, surface spotted, pale orange (5A3) to light brown (5D6). **Stipes** 1.6–5.4 × 0.1–0.3 cm, cylindrical, hallow, slightly enlarged at the base, strongly pruinose and slightly longitudinally striate, greyish orange (5B3) to light brown (5D6).

*Microscopic description:* **Basidiospores** (8.2)8.4–9.1(9.5) × (4.8)5.0–5.5(5.7) μm, Q = 1.59–1.74, Qm = 1.66 (±0.07), narrowly ellipsoid to oblong, some lentiform, with germ pore, slightly thin walled, contains oil droplets, orange–white (5A2) to greyish orange (5B4) in water, brownish orange (5C5) to yellowish brown (5E7) in 5% KOH. **Basidia** (14.6)16.1–19.5(21.8) × (6.6)7.6–10.0(11.1) μm, clavate, sometimes with vacuolar contents, two or four spored. **Cheilocystidia** (18.3)20.4–24.4(26.5) × (9.2)9.4–11.8(13.5) μm, lecythiform, with 5.3–7.2 μm wide capitula. **Pileipellis** hymeniform, consist of broadly clavate, obovoid, or spheropedunculate elements, (26.9)34.3–50.9(65.9) × (13.3)17.0–27.0(34.7) μm. **Pleurocystidia** utriform, broadly clavate or lecythiform, and rare in number. **Pileocystidia** not observed. **Clamp connections** present.

*Habit and habitat:* Summer scattered in the grassland. So far found only in the Xizang Autonomous Region, China.

*Notes:* In the phylogenetic analyses, *C. yadongensis* is closely related to *C. coniferarum* E.F. Malysheva, *C. echinata* (Velen.) Singer, and *C. qujingensis* W.H. Lu, Karunarathna & Tibpromma (Figure 1). However, *C. coniferarum* has longer stipe with brownish hues, broader basidiospores and four-spored basidia [22], while *C. echinata* has a dark brown to dull brown pileus and larger cheilocystidia [35,72]. Additionally, *C. qujingensis* has a longer stipe, larger cheilocystidia, and pileipellis consists of clavate and spheropedunculate elements [36].

## 4. Discussion

This study revisits the taxonomy of *Conocybe* specimens from the Q-X Plateau based on the phylogenetic frameworks established by Tóth et al. [21], Lu et al. [36], and Song and Bau [62], alongside the morphological classification by Hausknecht [34]. We incorporated macrofungal specimens collected from the Q-X Plateau between 2015 and 2024, and employed a concatenated dataset (including ITS, nrLSU, and *tef1-α* sequences) to reassess the classification of *Conocybe* in this region. Based on the results, we describe four new species: *C. alticola*, *C. alticoprophila*, *C. versicolor*, and *C. yadongensis*, from various locations, including the Ali Region, Shigatse Municipality, and Lingzhi Prefecture. These newly identified species expand the known diversity of *Conocybe* on the Q-X Plateau and offer preliminary insights into the species adaptation to high-altitude, cold-climate habitats. Additionally, there are three known species, *C. himalayana*, *C. pseudocrispa* (Hauskn.) Arnolds, and *C. fuscimarginata*, from Lhasa Municipality, Qamdo Municipality and the Ali Region. Notably, the latter two species are reported here for the first time from the Q-X Plateau. This study broadens the understanding of *Conocybe* diversity in this unique high-altitude region and provides foundational data on its distribution patterns in cold environments.

The formation of basidioma is closely influenced by environmental factors, such as temperature, light, and nutrients [73]. On the Q-X Plateau, most *Conocybe* taxa exhibit temperate characteristics, but *C. alticola*, which we have newly described and has tough basidioma and relatively short stipes, is an exception. This aligns with the toughness-protection hypothesis proposed by Krah et al., which suggests that tough-fleshed basidioma reduce water loss, helping macrofungi cope with extreme microclimatic fluctuations [74]. Despite extensive surveys across various regions of Hengduan Mountains and Xizang Autonomous Region, *C. alticola* has only been found in the Ali region of Xizang Autonomous Region, which is known for long daylight hours, low temperatures, intense sunlight, and high altitudes (with an average elevation exceeding 4500 m) [75,76].

Phylogenetically, *C. alticola* is clustered with *C. qujingensis*, *C. echinate*, and *C. coniferarum*, forming a distinct lineage, and is sister to *C. ammophila* (Figure 1), which is described as being found only in cold climates with extreme temperature fluctuations and low rainfall, such as Greenland, Mongolia, and Russia. It is one of the stoutest members of the genus [68], which aligns with the characteristics of *C. alticola*. In contrast, *C. qujingensis*, *C. echinata*, and *C. coniferarum* exhibit slender stipes and relatively fragile pileus [22,35,36]. The variation in macroscopic morphology partially reveals that species within the genus may undergo adaptive evolution changes when facing extreme environmental stress. To further substantiate this conclusion, additional sampling outside the current study area is required to explore the broader distribution of these species.

Currently, a total of 15 *Conocybe* species have been recorded from the Q-X Plateau, including *C. apala* (Fr.) Arnolds, *C. fragilis* (Peck) Singer, *C. himalayana*, *C. macrocephala* Kühner & Watling, *C. macrospora* (G.F. Atk.) Hauskn., *C. ochracea* (Kühner) J. Favre, *C. siliginea* (Fr.) Kühner, *C. subovalis* Kühner & Watling, *C. tenera* (Schaeff.) Kühner, and six species identified in this study [77,78,79,80,81,82,83,84,85]. In addition, *Conocybe* is primarily distributed in Jilin Province and Inner Mongolia, where 26 and 9 species have been reported, respectively [36]. Limited records have also been made in regions such as Guangxi Province, Hunan Province, Hubei Province, Yunnan Province, Taiwan, Tianjin Municipality, and Xinjiang Uygur Autonomous Region [19,57,77,78,85]. However, in comparison to the total number of known species, the diversity of *Conocybe* in China remains relatively underrepresented.

The classification of *Conocybe* remains challenging, primarily due to the discordance between the clades within genus and morphological sections, as well as the presence of morphologically similar species, which complicates accurate species identification [21,62]. Traditional identification relies on morphological features such as pileus, lamellae, stipe, which are influenced by environmental conditions and growth stages, leading to variation. Additionally, some species lack sufficient and reliable molecular data, making it difficult to conduct a phylogenetic analysis for accurate classification [36]. Therefore, a combined approach using multi-gene phylogenetic analysis and morphological characteristics, along with the inclusion of multidimensional regional samples, is essential for further clarifying the distribution and classification of this genus in China.

This study describes four new species and reports the first records of two known species from the Qinghai-Xizang Plateau. By combining morphological and molecular phylogenetic analyses, it provides preliminary insights into the adaptive evolution of macrofungi in extreme high-altitude cold climates, contributing new perspectives on fungal diversity and species distribution on the Plateau. It also offers valuable data for understanding species distribution patterns and environmental adaptations in high-altitude ecosystems and provides empirical support for the toughness-protection hypothesis.

## Figures and Tables

**Table 1:** Sequences used in the phylogenetic analysis in this study. Missing sequences are indicated by “–”.

Taxon Name	Voucher Number	ITS	nLSU	*tef1*	Country of Origin	References
*Conocybe* aff. *ochrostriata*	NL-0830	JX968236	JX968354	JX968447	Hungary	[54]
*Conocybe alboradicans*	NL-3226	JX968219	JX968336	JX968435	Hungary	[21]
*Conocybe alboradicans*	WU14678	JX968220	JX968337	–	Hungary	[54]
*Conocybe alkovii*	262841	JQ247196	–	–	Russia	[55]
***Conocybe alticola***	**ZRL20240256**	**PQ699268**	**PQ699292**	**PQ836630**	**China**	**This study**
***Conocybe alticola***	**ZRL20240259**	**PQ699269**	**PQ699293**	**PQ836631**	**China**	**This study**
***Conocybe alticola***	**ZRL20240316**	**PQ699270**	**PQ699294**	**PQ836632**	**China**	**This study**
***Conocybe alticola***	**ZRL20240317**	**PQ699271**	**PQ699295**	**PQ836633**	**China**	**This study**
***Conocybe alticola***	**ZRL20240327**	**PQ699272**	**PQ699296**	**PQ836634**	**China**	**This study**
***Conocybe alticoprophila***	**ZRL20220041**	**PQ699273**	**PQ699297**	**PQ836635**	**China**	**This study**
***Conocybe alticoprophila***	**ZRL20220046**	**PQ699274**	**PQ699298**	**PQ836636**	**China**	**This study**
***Conocybe alticoprophila***	**ZRL20220050**	**PQ699275**	**PQ699299**	**PQ836637**	**China**	**This study**
*Conocybe ammophila*	WU23983	JX968197	JX968313	JX968416	Mongolia	[19]
*Conocybe anthracophila*	WU14367	JX968212	JX968329	JX968430	Italy	[21]
*Conocybe anthracophila* var. *ovispora*	WU 25461	JX968237	JX968355	–	Italy	[19]
*Conocybe antipus*	WU19791	JX968215	JX968332	JX968432	Austria	[21]
*Conocybe apala*	NL-1012	JX968209	JX968326	JX968427	Hungary	[21]
*Conocybe aurea*	AMO16	MG663237	MT237453	–	USA	Unpublished
*Conocybe aurea*	WU28161	JX968184	JX968300	JX968407	Italy	[21]
*Conocybe bispora*	NL-2573	JX968203	JX968320	JX968423	Hungary	[21]
*Conocybe bisporigera*	HMJAU 45055	OP526418	–	–	China	[18]
*Conocybe bisporigera*	NL-1904	JX968235	JX968353	JX968446	Hungary	[19]
*Conocybe brachypodii*	HMJAU45017	MH141423	–	–	China	[19]
*Conocybe brachypodii*	NL-3169	JX968199	JX968316	JX968419	Hungary	[19]
*Conocybe ceracea*	HMJAU64951	OQ758110	OQ758218	OQ758305	China	[18]
*Conocybe ceracea*	HMJAU64952	OQ758111	OQ758219	OQ758306	China	[18]
*Conocybe ceracea*	HMJAU64953	OQ758112	OQ758220	–	China	[18]
*Conocybe cettoiana*	WU10436	JX968218	JX968335	–	Italy	[21]
*Conocybe confundens*	LE 313077	NR_155032	–	–	Russia	[22]
*Conocybe coniferarum*	LE 313009	NR_155030	–	–	Russia	[22]
*Conocybe crispella*	WU27367	JX968208	JX968325	JX968426	Austria	[21]
*Conocybe cylindracea*	WU 20796	JX968240	JX968358	JX968449	Italy	[21]
*Conocybe cylindrospora*	HMJAU42440	MG250375	OQ758203	–	China	[18,19]
*Conocybe cylindrospora*	HMJAU42441	MG250377	–	–	China	[19]
*Conocybe deliquescens*	HMJAU61998	OP373403	OQ758204	OQ758292	China	[18]
*Conocybe deliquescens*	NL-0574	JX968210	JX968327	JX968428	Hungary	[19]
*Conocybe dumetorum*	NL-2693	JX968201	JX968318	JX968421	Sweden	[21]
*Conocybe dunensis*	WU27359	JX968227	JX968345	–	Spain	[21]
*Conocybe echinata*	NL-1007	JX968196	JX968312	–	Hungary	[19]
*Conocybe elegans*	NL-0908	JX968223	JX968341	JX968437	Sweden	[21]
*Conocybe enderlei*	NL-0165	JX968161	JX968277	JX968389	Sweden	[21]
*Conocybe enderlei*	WU21272	JX968163	JX968279	–	Italy	[21]
*Conocybe farinacea*	NL-2173	JX968167	JX968283	–	Hungary	[21]
*Conocybe fiorii*	WU17793	JX968217	JX968334	JX968434	Italy	[21]
*Conocybe fuscimarginata*	HMJAU 45033	OQ780310	OQ758208	OQ758296	China	[18]
*Conocybe fuscimarginata*	NL-3668	JX968238	JX968356	JX968448	Sweden	[21]
***Conocybe fuscimarginata***	**ZRL20235165**	**PQ699276**	**PQ699300**	**PQ821725**	**China**	**This study**
***Conocybe fuscimarginata***	**ZRL20235225**	**PQ699277**	**PQ699301**	**PQ821726**	**China**	**This study**
*Conocybe gigasperma*	NL-3972	JX968179	JX968295	JX968403	Slovakia	[21]
*Conocybe graminis*	WU13466	JX968195	JX968311	–	Austria	[21]
*Conocybe hausknechtii*	LE253789	JQ247194	–	–	Russia	[56]
*Conocybe herbarum*	WU22193	JX968193	JX968309	–	Austria	[21]
*Conocybe himalayana*	HMAS 300530	PQ099839	PP968802	PP992938	China	[16]
*Conocybe himalayana*	HMAS 300531	PQ099840	PP968801	PP992937	China	[16]
*Conocybe himalayana*	HMAS 300532	PQ099841	PP968800	PP992939	China	[16]
*Conocybe himalayana*	HMAS 300534	PQ099842	PP968799	PP992936	China	[16]
*Conocybe himalayana*	HMAS 300535	PQ099843	PP968803	PP992940	China	[16]
***Conocybe himalayana***	**ZRL20231801**	**PQ699278**	**PQ699302**	**PQ821727**	**China**	**This study**
***Conocybe himalayana***	**ZRL20240260**	**PQ699279**	**PQ699303**	**PQ821728**	**China**	**This study**
***Conocybe himalayana***	**ZRL20240274**	**PQ699280**	**PQ699304**	**PQ821729**	**China**	**This study**
***Conocybe himalayana***	**ZRL20240279**	**PQ699281**	**PQ699305**	**PQ821730**	**China**	**This study**
***Conocybe himalayana***	**ZRL20240281**	**PQ699282**	**PQ699306**	**PQ821731**	**China**	**This study**
***Conocybe himalayana***	**ZRL20240283**	**PQ699283**	**PQ699307**	**PQ821732**	**China**	**This study**
***Conocybe himalayana***	**ZRL20240288**	**PQ699284**	**PQ699308**	**PQ821733**	**China**	**This study**
***Conocybe himalayana***	**ZRL20240318**	**PQ699285**	**PQ699309**	**PQ821734**	**China**	**This study**
***Conocybe himalayana***	**ZRL20240328**	**PQ699286**	**PQ699310**	**PQ821735**	**China**	**This study**
*Conocybe hornana*	NL-3499	JX968178	JX968294	JX968402	Slovakia	[21]
*Conocybe hydrophila*	HMJAU 64954	OQ758116	OQ758232	OQ758313	China	[18]
*Conocybe hydrophila*	HMJAU 64955	OQ758117	OQ758233	OQ758314	China	[18]
*Conocybe incarnata*	HMJAU 64968	OQ780316	–	–	China	[18]
*Conocybe incarnata*	WU 21897	JX968229	JX968347	JX968441	Finland	[21]
*Conocybe incerta*	LE 313017	NR_155031	–	–	Russia	[22]
*Conocybe ingridiae*	WU 28158	JX968244	JX968361	JX968451	Italy	[21]
*Conocybe inocybeoides*	NL-3589	JX968202	JX968319	JX968422	Hungary	[21]
*Conocybe inopinata*	WU27544	JX968165	JX968281	JX968392	Italy	[19]
*Conocybe intrusa*	WU25546	JX968211	JX968328	JX968429	Hungary	[21]
*Conocybe juniana*	NL-2105	JX968191	JX968307	JX968413	Hungary	[21]
*Conocybe juniana* var. *sordescens*	NL-2304	JX968192	JX968308	JX968414	Hungary	[21]
*Conocybe karakensis*	KTK-05	ON392730	–	–	Pakistan	[37]
*Conocybe karinae*	WU28526	JX968151	JX968268	JX968384	Hungary	[21]
*Conocybe lenticulospora*	HMJAU 45069	OQ780317	–	–	China	[18]
*Conocybe lenticulospora*	NL-0923	JX968242	JX968359	JX968450	Sweden	[21]
*Conocybe leporina*	NL-2380	JX968177	JX968293	JX968401	Hungary	[21]
*Conocybe lobauensis*	WU17826	JX968176	JX968292	JX968400	Italy	[19]
*Conocybe macrocephala*	WU18148	JX968182	JX968298	–	Austria	[19]
*Conocybe macrospora*	WU17030	JX968175	JX968291	–	Germany	[19]
*Conocybe merdaria*	WU25359	JX968174	JX968290	–	Austria	[19]
*Conocybe mesospora*	HMJAU45049	MH141419	–	–	China	[57]
*Conocybe microrrhiza*	NL-2180	JX968222	JX968340	JX968436	Hungary	[21]
*Conocybe microspora*	NL-1890	JX968160	JX968276	–	Hungary	[21]
*Conocybe monikae*	WU22612	JX968200	JX968317	JX968420	Hungary	[21]
*Conocybe moseri*	GLM-F 40421	MK412354	–	–	Germany	[18]
*Conocybe moseri*	HMJAU 45075	OQ780309	OQ758207	–	China	[18]
*Conocybe muscicola*	HMJAU64939	OQ758113	OQ758223	OQ758309	China	[18]
*Conocybe muscicola*	HMJAU64940	OQ758115	OQ758224	OQ758310	China	[18]
*Conocybe nigrescens*	WU 27557	JX968234	JX968352	JX968445	Italy	[21]
*Conocybe nitrophila*	WANG 140019	KR998384	–	–	China	[58]
*Conocybe nitrophila*	WU20916	JX968233	JX968351	JX968444	India	[21]
*Conocybe ochrostriata* var. *favrei*	WU 29786	JX968245	JX968362	JX968452	Italy	[21]
*Conocybe olivaceopileata*	LE 313106	NR_155028	–	–	Russia	[22]
*Conocybe pallidospora*	WU 7395	JX968239	JX968357	–	Austria	[21]
*Conocybe pallidospora*	WU17079	JX968158	–	–	Hungary	[21]
*Conocybe papillata*	NL-2370	JX968216	JX968333	JX968433	Hungary	[21]
*Conocybe parapilosella*	90551	NR_176713	–	–	Spain	[59]
*Conocybe parapilosella*	WU30224	MN872706	–	–	Spain	[59]
*Conocybe pilosa*	HMJAU 64947	OQ758122	OQ758222	OQ758307	China	[18]
*Conocybe pilosa*	HMJAU 64948	OQ758123	OQ758221	OQ758308	China	[18]
*Conocybe pilosella*	HMJAU 45062	OQ780305	OQ758205	OQ758294	China	[18]
*Conocybe pilosella*	HMJAU 64957	OQ780306	OQ758206	OQ758295	China	[18]
*Conocybe praticola*	HMJAU64965	OQ780303	–	–	China	[18]
*Conocybe praticola*	LE 313010	KY614060	–	–	Russia	[22]
*Conocybe pseudocrispa*	HMJAU 64944	OQ780308	OQ758211	OQ758298	China	[18]
*Conocybe pseudocrispa*	WU 18009	JX968230	JX968348	JX968442	Austria	[21]
***Conocybe pseudocrispa***	**ZRL20240311**	**PQ699287**	**PQ699311**	**PQ821736**	**China**	**This study**
***Conocybe pseudocrispa***	**ZRL20240319**	**PQ699288**	**PQ699312**	**PQ821737**	**China**	**This study**
*Conocybe pubescens*	NL-1986	JX968173	JX968289	JX968399	Hungary	[21]
*Conocybe pubescens*	WU20759	JX968170	JX968286	JX968396	Italy	[21]
*Conocybe punjabensis*	SKP066	MH981969	–	–	Pakistan	[54]
*Conocybe punjabensis*	SKP069	MH981968	–	–	Pakistan	[54]
*Conocybe qujingensis*	HKAS 127149	OQ755415	–	–	China	[36]
*Conocybe qujingensis*	HKAS 128155	OQ755416	–	–	China	[36]
*Conocybe qujinguniversitatis*	HKAS 128150	OQ755413	–	–	China	[36]
*Conocybe qujinguniversitatis*	HKAS 128151	OQ755414	–	–	China	[36]
*Conocybe reniformis*	HMJAU 64942	OQ758108	OQ758229	OQ758311	China	[18]
*Conocybe reniformis*	HMJAU 64943	OQ758109	OQ758228	OQ758312	China	[18]
*Conocybe rickeniana*	NL-2468	JX968198	JX968315	JX968418	Hungary	[51]
*Conocybe rickenii*	AH 21067	MF142238	–	–	Spain	[60]
*Conocybe romagnesii*	WU26605	JX968206	JX968323	JX968424	Italy	[19]
*Conocybe rostellata*	NL-2499	JX968162	JX968278	JX968390	Sweden	[21]
*Conocybe rufostipes*	HMJAU 64937	OQ758120	OQ758227	OQ758317	China	[18]
*Conocybe rufostipes*	HMJAU 64938	OQ758121	OQ758226	OQ758318	China	[18]
*Conocybe sabulicola*	WU11185	JX968186	JX968302	JX968409	Italy	[19]
*Conocybe semiglobata*	NL-1993	JX968181	JX968297	JX968405	Hungary	[19]
*Conocybe semiglobata*	WU8794	JX968188	JX968304	–	Austria	[21]
*Conocybe semiglobata* var. *campanulata*	NL-3159	JX968168	JX968284	JX968394	Austria	[21]
*Conocybe semiglobata* var. *campanulata*	WU26395	JX968169	JX968285	JX968395	Germany	[19]
*Conocybe siennophylla*	HMJAU 64966	OQ780312	OQ758210	OQ758297	China	[18]
*Conocybe siennophylla*	NL-1210	JX968246	JX968363	JX968453	Hungary	[21]
*Conocybe siennophylla*	WU17988	JX968243	JX968360	–	Hungary	[21]
*Conocybe siliginea*	NL-1211	JX968159	JX968275	–	Sweden	[21]
*Conocybe siliginea*	NL-2313	JX968225	JX968343	JX968438	Sweden	[21]
*Conocybe singeriana*	WU22129	JX968166	JX968282	JX968393	Austria	[21]
*Conocybe sinobispora*	HMJAU 64949	OQ758118	OQ758230	OQ758315	China	[18]
*Conocybe sinobispora*	HMJAU 64950	OQ758119	OQ758231	OQ758316	China	[18]
*Conocybe solitaria*	WU20903	JX968214	JX968331	JX968431	China	[21]
*Conocybe* sp.	HMJAU44988	OQ749737	–	OQ758302	China	[18]
*Conocybe* sp.	HMJAU64962	OQ749739	–	OQ758303	China	[18]
*Conocybe* sp.	HMJAU64967	OQ749741	–	–	China	[18]
*Conocybe* sp.	HMJAU64964	OQ749742	–	–	China	[18]
*Conocybe* sp.	HMJAU44994	OQ749735	–	–	China	[18]
*Conocybe* sp.	NL-1455	JX968194	JX968310	JX968415	Hungary	[21]
*Conocybe subovalis*	NL-1415	JX968190	JX968306	JX968412	Hungary	[19]
*Conocybe subpubescens*	NL-0162	JX968189	JX968305	JX968411	Sweden	[19]
*Conocybe subpubescens*	NL-2181	JX968171	JX968287	JX968397	Hungary	[19]
*Conocybe subxerophytica*	NL-0164	JX968187	JX968303	JX968410	Sweden	[19]
*Conocybe tenera*	NL-	JX968185	JX968301	JX968408	Hungary	[21]
*Conocybe tenera*	NL-1615	JX968180	JX968296	JX968404	Hungary	[19]
*Conocybe tetrasporoides*	WU 17385	JX968232	JX968350	–	New Zealand	[21]
*Conocybe turkestanica*	H 7034981	MH055382	–	–	Turkestan	[19]
*Conocybe tuxlaensis*	NL-1897	JX968164	JX968280	JX968391	Hungary	[21]
*Conocybe vaginata*	WU25703	JX968204	JX968321	–	Sri Lanka	[21]
*Conocybe velutinomarginata*	WU 28695	JX968226	JX968344	JX968439	Germany	[21]
*Conocybe velutipes*	HMJAU 45048	OQ780311	OQ758209	–	China	[18]
*Conocybe velutipes*	NL-2187	JX968228	JX968346	JX968440	Hungary	[21]
***Conocybe versicolor***	**ZRL20152517**	**PQ699289**	**PQ699313**	**PQ836638**	**China**	**This study**
***Conocybe versicolor***	**ZRL20220299**	**PQ699290**	**PQ699314**	**PQ836639**	**China**	**This study**
*Conocybe volvata*	WU20900	JX968205	JX968322	–	India	[19]
*Conocybe volvicystidiata*	1212	KY346827	–	–	France	[61]
*Conocybe watlingi*	WU22744	JX968172	JX968288	JX968398	Finland	[21]
***Conocybe yadongensis***	**ZRL20220042**	**PQ699291**	**PQ699315**	**PQ836640**	**China**	**This study**
*Conocybe zeylanica*	WU20185	JX968207	JX968324	JX968425	La Re’union	[21]
*Conocybe zuccherellii*	WU12421	JX968213	JX968330	–	Italy	[21]
*Bolbitius coprophilus*	HMJAU64958	OQ780315	OQ758216	–	China	[18]
*Bolbitius reticulatus*	WU30001	JX968249	JX968366	JX968455	Hungary	[21]
*Bolbitius subvolvatus*	WU28379	JX968248	JX968365	JX968454	Italy	[21]
*Conobolbitina micheliana*	HMJAU65015	OR995677	OR994080	PP000869	China	[62]
*Conobolbitina micheliana*	HMJAU65016	OR995678	OR994081	PP000870	China	[62]
*Conobolbitina ochroleuca*	HMJAU65017	OR995679	OR994082	PP000871	China	[62]
*Conobolbitina ochroleuca*	HMJAU65018	OR995680	OR994083	PP000872	China	[62]
*Conocybula coprophila*	SZMC-NL-2176	JX968156	JX968273	–	Hungary	[21]
*Conocybula cyanopus*	WU2134	JX968157	JX968274	JX968388	Austria	[21]
*Galerella nigeriensis*	CNF1/5859	JX968251	JX968368	JX968457	Nigeria	[21]
*Descolea antarctica*	NZ5182	AF325647	–	–	USA	[63]
*Descolea quercina*	HMJAU64959	OQ780313	OQ758213	OQ758299	China	[18]
*Pholiotina changbaishanensis*	HMJAU65101	OR995689	OR994092	PP000881	China	[62]
*Pholiotina changbaishanensis*	HMJAU65102	OR995690	OR994093	PP000882	China	[62]
*Pholiotina serrata*	HMJAU42442	MG250376	–	–	China	[19]
*Pholiotina serrata*	HMJAU62006	OP538570	OQ758217	OQ758301	China	[18]
*Pholiotina eburnea*	HMJAU65034	OR995693	OR994096	PP000885	China	[62]
*Pholiotina eburnea*	HMJAU65035	OR995694	OR994097	PP000886	China	[62]
*Pholiotina excrescenticystidiata*	HMJAU65021	OR995695	OR994098	PP000887	China	[21]
*Pholiotina excrescenticystidiata*	HMJAU65022	OR995696	OR994099	PP000888	China	[62]
*Pholiotina pleurocystidiata*	WU40666	NR_176740	–	–	Austria	[64]
*Candolleomyces sichuanicus*	HMAS 287616	PP734617	PP734628	PP729330	China	[46]
*Candolleomyces sichuanicus*	HMAS 287617	PP734618	PP734629	PP729331	China	[46]
*Candolleomyces singeri*	HMJUA37867	MG734718	MW301088	MW314077	China	[65]
*Candolleomyces singeri*	HMJAU37877	MW301073	MW301091	MW314080	China	[11]
*Psathyrella piluliformis*	HMJAU37922	MG734716	MW413364	MW411001	China	[65]
*Psathyrella amygdalinospora*	HMJAU37952	MW405104	MW413361	MW410999	China	[66]
*Psathyrella amygdalinospora*	HMJAU57044	MW405105	–	–	China	[66]

The sequences generated in this study are marked in bold.

## Data Availability

All sequence data are available in NCBI GenBank following the accession numbers in the manuscript.

## References

[1] Zhu Q., Chen H., Peng C., Liu J., Piao S., He J.-S., Wang S., Zhao X., Zhang J., Fang X. (2023). An early warning signal for grassland degradation on the Qinghai-Tibetan Plateau. Nat. Commun..

[2] Mao K.S., Wang Y., Liu J.Q. (2021). Evolutionary origin of species diversity on the Qinghai–Tibet Plateau. J. Syst. Evol..

[3] Renner S.S. (2016). Available data point to a 4-km-high Tibetan Plateau by 40 Ma, but 100 molecular-clock papers have linked supposed recent uplift to young node ages. J. Biogeogr..

[4] Yang M.X., Werth S., Wang L.S., Scheidegger C. (2022). Phylogeographic analyses of an epiphytic foliose lichen show multiple dispersal events westward from the Hengduan Mountains of Yunnan into the Himalayas. Ecol. Evol..

[5] Yu H., Deane D.C., Sui X., Fang S., Chu C., Liu Y., He F. (2019). Testing multiple hypotheses for the high endemic plant diversity of the Tibetan Plateau. Glob. Ecol. Biogeogr..

[6] Ding L., Kapp P., Cai F., Garzione C.N., Xiong Z., Wang H., Wang C. (2022). Timing and mechanisms of Tibetan Plateau uplift. Nat. Rev. Earth Environ..

[7] Deng T., Wu F., Zhou Z., Su T. (2020). Tibetan Plateau: An evolutionary junction for the history of modern biodiversity. Sci. China Earth Sci..

[8] Liu H., Wang W., Song G., Qu Y., Li S.-H., Fjeldså J., Lei F. (2012). Interpreting the process behind endemism in China by integrating the phylogeography and ecological niche models of the *Stachyridopsis ruficeps*. PLoS ONE.

[9] Yang T., Adams J.M., Shi Y., He J.S., Jing X., Chen L., Tedersoo L., Chu H. (2017). Soil fungal diversity in natural grasslands of the Tibetan Plateau: Associations with plant diversity and productivity. New Phytol..

[10] Zhang T., Wang N., Yu L. (2020). Soil fungal community composition differs significantly among the Antarctic, Arctic, and Tibetan Plateau. Extremophiles.

[11] Li Y., Zhou Y., Liu F., Liu X., Wang Q. (2022). Diversity patterns of wetland angiosperms in the Qinghai-Tibet Plateau, China. Diversity.

[12] Li Q., He G., Wen T., Zhang D., Liu X. (2022). Distribution pattern of soil fungi community diversity in alpine meadow in Qilian Mountains of eastern Qinghai-Tibetan Plateau. Ecol. Indic..

[13] Phurbu D., Huang J.-E., Song S., Ni Z., Zhou X., Li S., Cai L., Liu F. (2024). Diversity of culturable fungi in six Tibetan Plateau lakes, with descriptions of eight new taxa. Mycology.

[14] Yang N., Li X., Liu D., Zhang Y., Chen Y., Wang B., Hua J., Zhang J., Peng S., Ge Z. (2022). Diversity patterns and drivers of soil bacterial and fungal communities along elevational gradients in the Southern Himalayas, China. Appl. Soil Ecol..

[15] Han X., Liu D., Zhang M., He M., Li J., Zhu X., Wang M., Thongklang N., Zhao R., Cao B. (2023). Macrofungal diversity and distribution patterns in the primary forests of the shaluli mountains. J. Fungi.

[16] Wang K., Liu S.-L., Liu X.-Z., Hong P., Wei H.-W., Wang Y., Phurbu D., Zhou L.-W., Wei T.-Z. (2024). Catalogue of fungi in China 3. New taxa of macrofungi from southern Xizang, China. Mycology.

[17] He M.-Q., Cao B., Liu F., Boekhout T., Denchev T.T., Schoutteten N., Denchev C.M., Kemler M., Gorjón S.P., Begerow D. (2024). Phylogenomics, divergence times and notes of orders in Basidiomycota. Fungal Divers..

[18] Song H.-B., Bau T. (2023). *Conocybe* section *Pilosellae* in China: Reconciliation of taxonomy and phylogeny reveals seven new species and a new record. J. Fungi.

[19] Liu J., Bau T. (2018). New species and new records in the genus *Conocybe* (Bolbitaceae) from China. Phytotaxa.

[20] Amandeep K., Atri N., Munruchi K. (2015). Diversity of species of the genus *Conocybe* (Bolbitiaceae, Agaricales) collected on dung from Punjab, India. Mycosphere.

[21] Tóth A., Hausknecht A., Krisai-Greilhuber I., Papp T., Vágvölgyi C., Nagy L.G. (2013). Iteratively refined guide trees help improving alignment and phylogenetic inference in the mushroom family Bolbitiaceae. PLoS ONE.

[22] Malysheva E.F. (2017). Five new species of *Conocybe* (Agaricomycetes, Bolbitiaceae) from Russia. Mycol. Prog..

[23] He M.Q., Wang M.Q., Chen Z.H., Deng W.Q., Li T.H., Vizzini A., Jeewon R., Hyde K.D., Zhao R.L. (2022). Potential benefits and harms: A review of poisonous mushrooms in the world. Fungal Biol. Rev..

[24] Pu C.-J., Peng Y.-L., Li Z.-H., He J., Huang R., Feng T., Liu J.-K. (2019). Two highly oxygenated ergosterols from cultures of the basidiomycete *Conocybe siliginea*. Nat. Prod. Res..

[25] Luo H., Hallen-Adams H.E., Walton J.D. (2009). Processing of the phalloidin proprotein by prolyl oligopeptidase from the mushroom *Conocybe albipes*. J. Biol. Chem..

[26] He J., Pu C.-J., Wang M., Li Z.-H., Feng T., Zhao D.-K., Liu J.-K. (2020). Conosiligins A–D, ring-rearranged tremulane sesquiterpenoids from *Conocybe siliginea*. J. Nat. Prod..

[27] Erritzoe D., Barba T., Greenway K.T., Murphy R., Martell J., Giribaldi B., Timmermann C., Murphy-Beiner A., Jones M.B., Nutt D. (2024). Effect of psilocybin versus escitalopram on depression symptom severity in patients with moderate-to-severe major depressive disorder: Observational 6-month follow-up of a phase 2, double-blind, randomised, controlled trial. eClinicalMedicine.

[28] Griffiths R.R., Johnson M.W., Carducci M.A., Umbricht A., Richards W.A., Richards B.D., Cosimano M.P., Klinedinst M.A. (2016). Psilocybin produces substantial and sustained decreases in depression and anxiety in patients with life-threatening cancer: A randomized double-blind trial. J. Psychopharmacol..

[29] Wu F., Zhou L.W., Yang Z.L., Bau T., Li T.H., Dai Y.C. (2019). Resource diversity of Chinese macrofungi: Edible, medicinal and poisonous species. Fungal Divers..

[30] Hallen H.E., Watling R., Adams G.C. (2003). Taxonomy and toxicity of *Conocybe lactea* and related species. Mycol. Res..

[31] Matheny P.B., Curtis J.M., Hofstetter V., Aime M.C., Moncalvo J.-M., Ge Z.-W., Yang Z.-L., Slot J.C., Ammirati J.F., Baroni T.J. (2006). Major clades of Agaricales: A multilocus phylogenetic overview. Mycologia.

[32] Hausknecht A., Contu M. (2007). Interesting species of *Conocybe* (Agaricales, Bolbitiaceae) from Gallura (NE Sardinia, Italy). Österr. Z. Pilzk.

[33] Watling R. (1971). The genus *Conocybe subgenus* Pholiotina II. Some European exannulate species and North American annulate species. Persoonia-Mol. Phylogeny Evol. Fungi..

[34] Hausknecht A. (2009). A monograph of the genera Conoybe Fayod, Pholiotina Fayod in Europe. Fungi Eur..

[35] Prydiuk M.P. (2014). Some rare and interesting *Conocybe* found in Vyzhnytsia National Nature Park (Ukrainian Carpathians). Mycobiota.

[36] Lu W., Suwannarach N., Lumyong S., Elgorban A.M., Dai D.-Q., Dutta A.K., Han L.-H., Tibpromma S., Karunarathna S.C. (2024). Molecular phylogeny and morphology reveal two new species of *Conocybe* (Bolbitiaceae, Agaricales) from southwest China. N. Z. J. Bot..

[37] Ullah T., Ullah K., Saba M., Shah F.H. (2023). *Conocybe karakensis* sp. nov.(Bolbitiaceae, Agaricales) from Pakistan. Phytotaxa.

[38] Largent D. (1986). How to Identify Mushrooms to Genus Vol. I. Macroscopic Features.

[39] Bau T., Yan J.Q. (2021). Two new rare species of *Candolleomyces* with pale spores from China. MycoKeys.

[40] Kornerup A., Wanscher J.H. (1967). Methuen Handbook of Colour.

[41] Gardes M., Bruns T.D. (1993). ITS primers with enhanced specificity for basidiomycetes-application to the identification of mycorrhizae and rusts. Mol. Ecol..

[42] Hopple Jr J.S., Vilgalys R. (1999). Phylogenetic relationships in the mushroom genus *Coprinus* and dark-spored allies based on sequence data from the nuclear gene coding for the large ribosomal subunit RNA: Divergent domains, outgroups, and monophyly. Mol. Phylogenetics Evol..

[43] Örstadius L., Ryberg M., Larsson E. (2015). Molecular phylogenetics and taxonomy in Psathyrellaceae (Agaricales) with focus on psathyrelloid species: Introduction of three new genera and 18 new species. Mycol. Prog..

[44] Li J.X., Cao B., Phurbu D., He M.Q., Zhu X.Y., Parra L.A., Zhao R.L. (2024). The revision of the taxonomic system of Lycoperdaceae. Mycosphere.

[45] Mou G.-F., Bau T. (2021). Asproinocybaceae fam. nov. (Agaricales, Agaricomycetes) for Accommodating the Genera *Asproinocybe* and *Tricholosporum*, and Description of *Asproinocybe sinensis* and *Tricholosporum guangxiense* sp. nov. J. Fungi.

[46] Han X.X., Phurbu D., Ma G.F., Li Y.Z., Mei Y.J., Liu D.M., Lin F.C., Zhao R.L., Thongklang N., Cao B. (2024). A Taxonomic Study of *Candolleomyces* Specimens from China Revealed Seven New Species. J. Fungi.

[47] Edgar R.C. (2004). MUSCLE: Multiple sequence alignment with high accuracy and high throughput. Nucleic Acids Res..

[48] Hall T.A. (1999). BioEdit: A user-friendly biological sequence alignment editor and analysis program for Windows 95/98/NT. Nucleic Acids Symp. Ser..

[49] Zhang D., Gao F., Jakovlić I., Zou H., Zhang J., Li W.X., Wang G.T. (2020). PhyloSuite: An integrated and scalable desktop platform for streamlined molecular sequence data management and evolutionary phylogenetics studies. Mol. Ecol. Resour..

[50] Silvestro D., Michalak I. (2012). raxmlGUI: A graphical front-end for RAxML. Org. Divers. Evol..

[51] Lanfear R., Frandsen P.B., Wright A.M., Senfeld T., Calcott B. (2017). PartitionFinder 2: New methods for selecting partitioned models of evolution for molecular and morphological phylogenetic analyses. Mol. Biol. Evol..

[52] Ronquist F., Teslenko M., Van Der Mark P., Ayres D.L., Darling A., Höhna S., Larget B., Liu L., Suchard M.A., Huelsenbeck J.P. (2012). MrBayes 3.2: Efficient Bayesian phylogenetic inference and model choice across a large model space. Syst. Biol..

[53] Letunic I., Bork P. (2024). Interactive Tree of Life (iTOL) v6: Recent updates to the phylogenetic tree display and annotation tool. Nucleic Acids Res..

[54] Izhar A., Bashir H., Khalid A.N. (2019). A new species of *Conocybe* (Bolbitaceae) from Punjab, Pakistan. Phytotaxa.

[55] Malysheva E. (2012). *Conocybe* (Bolbitiaceae, Agaricomycetes) in the Russian Far East: New species and new section. Микoлoгия И Фитoпатoлoгия.

[56] Malysheva E.F. (2013). *Conocybe hausknechtii*, a new species of sect. *Pilosellae* from the Western Caucasus, Russia. Mycotaxon.

[57] Liu J. (2018). Taxonomy and Molecular Phylogeny of Bolbitiaceae in Northeast China.

[58] Wang Y.-W., Tzean S.-S. (2015). Dung-associated, potentially hallucinogenic mushrooms from Taiwan. Taiwania.

[59] Siquier J., Salom J. (2021). Contributo alla conoscenza dei Generi *Conocybe* (II) e *Pholiotina* (II) delle Isole Baleari (Spagna). *Conocybe parapilosella* sp. nov. Riv. Micol..

[60] Siquier J., Salom J. (2018). *Contributo alla* conescenza del genere *Conocybe nelle* Isole Baleari (Spagna). I. Riv. Di Micol..

[61] Hausknecht A., Broussal M. (2016). *Conocybe volvicystidiata*, a new species of the section Singerella. Osterr. Z. Für Pilzkd..

[62] Song H.B., Bau T. (2024). Resolving the polyphyletic origins of *Pholiotina* s.l. (Bolbitiaceae, Agaricales) based on Chinese materials and reliable foreign sequences. Mycosphere.

[63] Peintner U., Bougher N.L., Castellano M.A., Moncalvo J.M., Moser M.M., Trappe J.M., Vilgalys R. (2001). Multiple origins of sequestrate fungi related to *Cortinarius* (Cortinariaceae). Am. J. Bot..

[64] Hausknecht A., Krisai-Greilhuber I. (2020). *Pholiotina pleurocystidiata* (Bolbitiaceae), eine neue Art mit Pleuro-zystiden Pholiotina pleurocystidiata (Bolbitiaceae), a new species with pleuro-cystidia. Osterr. Z. Für Pilzkd..

[65] Yan J.Q., Bau T. (2018). The Northeast Chinese species of *Psathyrella* (Agaricales, Psathyrellaceae). MycoKeys.

[66] Bau T., Yan J.-Q. (2021). A new genus and four new species in the/*Psathyrella* sl clade from China. MycoKeys.

[67] Hausknecht A., Krisai-Greilhuber I., Voglmayr H. (2004). Type studies in north American species of Bolbitiaceae belonging to the genera *Conocybe* and *Pholiotina*. Osterr. Z. Für Pilzkd..

[68] Hausknecht A., Kalamees K., Knudsen H., Mukhin V. (2009). The genera *Conocybe* and *Pholiotina* (agaricomycotina, Bolbitiaceae) in temperate Asia. Folia Cryptogam. Est..

[69] Karun N., Sridhar K. (2015). Elephant dung-inhabiting macrofungi in the Western Ghats. Curr. Res. Environ. Appl. Mycol..

[70] Hausknecht A. (1996). Beitrage zur kenntnis der Bolbitiaceae 3. Europaische *Conocybe*-*arten* mit wurzelndem oder tief im substrat eingesenktem stiel. Osterr Z Pilzk..

[71] Thomas K.A., Hausknecht A., Manimohan P. (2001). Bolbitiaceae of Kerala State, India: New species and new and noteworthy records. Osterr. Z. Für Pilzkd..

[72] Prydiuk M.P. (2007). New records of *Conocybe* species from Ukraine. II. The section *Conocybe*. Czech Mycol..

[73] Sakamoto Y. (2018). Influences of environmental factors on fruiting body induction, development and maturation in mushroom-forming fungi. Fungal Biol. Rev..

[74] Krah F.S., Hagge J., Schreiber J., Brandl R., Müller J., Bässler C. (2022). Fungal fruit body assemblages are tougher in harsh microclimates. Sci. Rep..

[75] Sun X.Y., Miao L.Z., Feng X.K., Zhan X.X. (2024). Snow Disaster Risk Assessment Based on Long-Term Remote Sensing Data: A Case Study of the Qinghai-Tibet Plateau Region in Xizang. Remote Sens..

[76] Kong H.J., Wang J.G., Cai L., Cao J.X., Zhou M., Fan Y.D. (2024). Surface Solar Radiation Resource Evaluation of Xizang Region Based on Station Observation and High-Resolution Satellite Dataset. Remote Sens..

[77] Mao X.L. (1998). Economic Fungi of China.

[78] Bau T., Li Y. (2000). Study on fungal flora diversity in Daqinggou Nature Reserve. Biodivers. Sci..

[79] Mao X.L. (1985). Alpine macrofungi of East Himalaya and their adaptive characteristics. Mt Res..

[80] Mao X.L. (1985). The resources of macrofungi from the Mt. Namjagbarwa region in Xizang (Tibet), China. Acta Mycol. Sin..

[81] Mao X.L. (2000). The Macrofungi in China.

[82] Yuan M.S., Sun P.Q. (1995). Mushrooms of Sichuan.

[83] Bau T., Li Y. (2001). Notes on Fungi in Tibet of China. Bull. Bot. Res..

[84] Yuan M., Sun P. (2007). The Pictorial Book of Mushrooms of China.

[85] Zhuang W.Y. (2005). Fungi of Northwestern China.

